# Enhancing the Bioavailability of Poorly Soluble Drugs

**DOI:** 10.3390/pharmaceutics16060758

**Published:** 2024-06-04

**Authors:** Rodolfo Pinal

**Affiliations:** Department of Industrial and Molecular Pharmaceutics, Purdue University, West Lafayette, IN 47907, USA; rpinal@purdue.edu

The poor aqueous solubility of drugs is such a frequent challenge to drug absorption, bioavailability, and drug delivery that it is occasionally spoken about as an “unwritten rule” in drug product development. Fortunately, the long-standing solubility challenge continues to inspire novel formulation, processing, and screening strategies aimed at enhancing the bioavailability of poorly soluble drugs. In this Special Issue, a contribution by Tedesco et al. presents an illustrative case pointing toward opportunities for formulation refinements to optimize the relationship between drug concentration, as released by the formulation (bioaccessibility), and bioavailability, even for a recently developed commercial product, which necessarily, has been previously shown to be safe and therapeutically effective.

In this Special Issue, readers will find a cross-section of state-of-the-art articles, including three reviews, showcasing the latest advances in methodologies developed for addressing the repercussions of the poor solubility problem of drugs. Topics covered in this issue range from salt formation/selection to amorphous formulations, self-emulsifying systems, lipid-based formulations, nanoparticle-based systems, new materials, novel uses of old materials, innovations in screening technologies for dissolution and tissue uptake, and pharmaceutical processing.

Amorphous formulations and self-emulsifying systems have been subjects of intense research activity in the recent past, and this trend continues, as evidenced in this Special Issue. The use of amorphous solid dispersions (ASDs) has long been established as an effective method for enhancing drug solubility and improving bioavailability. Formulation scientists recognize that the key to the success of ASDs largely depends on optimizing the process and materials involved, based on the performance desired for the specific product. Such optimization is a multi-pronged effort involving materials and composition, as well as processing methods and conditions. In their article, Lee et al. show how a carefully designed strategy aims at maintaining physical stability while simultaneously maximizing drug dissolution rate, achievable degree of supersaturation, and enhancement of permeability, with each attribute contributing toward improvements in bioavailability, and the combination yielding more than the sum of its parts. The article contributed by Dharani et al. reveals that ASDs can be further enhanced in terms of their performance through additional processes such as solvent evaporation methods, without incurring the potential stability issues associated with the high temperatures used with hot-air drying or hot-melt extrusion. Moreover, as Xu et al. report in their article, amorphous systems are being developed, with researchers taking advantage of the energy of intermolecular interactions. Namely, the crystal lattice of a drug is disrupted via complex formation, which, being a spontaneous process, offers inherent stability advantages. In their report, Abdelkader et al. take the utility and application of amorphous formulations beyond ASDs. Specifically, amorphous formulations can also be realized as film matrices, which are utilized to render the drug amorphous. These films can be then processed into granules to produce compressed tablets.

On the general subject of self-emulsifying systems, new methods of preparing these drug delivery formulations have emerged, notably through the use of hot-melt extrusion. As demonstrated in the contribution by Zupančič et al., this methodology addresses both the hydrophobicity and lattice energy challenges associated with poor drug solubility. We find another self-emulsifying methodology in the contribution by Choi et al., namely, solution chemistry phenomena are employed to optimize self-emulsifying formulations, where multi-component liquid mixtures are used to maximize drug solubilization while minimizing particle size upon self-emulsification. In their article, Kovačević et al. take self-emulsifying systems further. Innovative processing approaches in their work include the integration of various elements, such as microporous matrices, in self-emulsifying systems, facilitated by high-shear granulation. This method exemplifies a formulation approach that merges aspects from both amorphous and lipid formulations, aiming to overcome both solid- and solution-phase solubility limitations simultaneously.

Drug absorption and ensuing bioavailability, in simplistic terms, result from the combined effects of (1) the ability of a formulation to solubilize the drug and (2) the response of the tissue when exposed to the drug. Mohammadi-Meyabadi et al. report on new tools for evaluating drug uptake in different tissues. Among various absorption sites, the oral mucosa has received attention as a drug delivery site for patients, notably for its practical convenience and its ability to bypass first-pass metabolism. The maximum drug absorption from the oral mucosa depends on the optimization of uptake by the oral tissue for the specific drug. The review article by He and Mu thoroughly covers the strategic use of pH modification, leading to significantly improved buccal and sublingual formulations, exhibiting enhanced permeability and bioavailability. Furthermore, understanding the interaction between tissue and drug for either different APIs or for different regions of the gastrointestinal (GI) tract is crucial, and this Special Issue highlights ongoing efforts to utilize biocompatible polymer systems, which take advantage of ionic interactions to form structured bodies. In another review article included in this Special Issue, the contribution by Gadziński et al., readers are provided thorough information on how these types of polymer-based systems can be further engineered through gelation and cross-linking techniques, to overcome the limitations of traditional oral dosage forms delivered to the GI tract.

The majority of drugs approved in the US, administered in solid form, are marketed in salt form. Salt formation is a well-established means of increasing drug solubility. Advances in salt identification/selection have evolved into the field of solid crystal forms. The article by Yu et al. illustrates how the same drug–counterion pair can produce a variety of solid salt forms, extending the range of possibilities for the salt formation/selection approach.

Innovative materials are aiding in the advancement of pharmaceutical product development. The article by Xiong et al. details how cross-linked metal–organic frameworks utilizing cyclodextrins are being applied to enhance the targeted uptake of chemotherapeutic agents in local tissues. Furthermore, in the article contributed by Song et al., new types of materials are identified, namely organic–mineral matrices like lipid dispersions formulated with clay material, which provide stability and an enhanced dissolution profile. In the review article contributed by V. Ambrogi, we learn about new developments in the functionality of an old material. Calcium carbonate, typically used as a bulking agent with pH-responsive properties, is now considered from a new perspective. By leveraging its different crystal forms, this “old” excipient is finding novel applications, such as serving as a matrix for drug molecules. These matrices have the valuable functionality of enhancing drug bioavailability.

Solvent screening is an integral part of formulation development, especially for poorly soluble drugs, as its importance is not limited to the understanding of the solubility properties of the drug. Solvent screening is crucial to the development of drug release tests capable of discriminating among various formulation and processing approaches, including particle size reduction, amorphous systems, and salt selection, among others. The goal is to help identify and/or optimize the most suitable formulation and manufacturing process. While this type of work is essential, it is also recognized as being time- and resource-intensive. To address these issues, Cysewski et al. report on the application of deep machine learning to maximize the quality of the information obtained during solvent selection, simultaneously reducing the time and resources employed. Furthermore, formal dissolution methods, which are quite useful in assessing product quality, have been enhanced through novel modifications. In the article contributed by Katona et al., researchers show that by reconfiguring the dissolution equipment into a gastrointestinal simulator, the modified dissolution testing system improves the ability to assess and compare not only the quality and reproducibility, but also the performance, of oral solid dosage forms.

Nanotechnology represents another crucial area in tackling the formulation challenges posed by poor drug solubility, particularly through the use of nanoscale delivery systems like liposomes. These have proven effective in encapsulating drugs, and recent advances include modifying the surface of liposomes with a biopolymer coating. This type of refinement, reported by Homayoonfal et al. in this Special Issue, enhances the drug-carrying capacity of liposomes, thus allowing for regulated drug delivery performance. Additionally, silk fibroin nanoparticles (SFNs) have demonstrated great usefulness in enhancing the bioavailability of poorly soluble drugs. However, a factor that limits the widespread use of SFNs is their time-consuming preparation process, which also faces issues in terms of reproducibility and scalability. The article contributed by Ruiz-Alcaraz et al. addresses this dilemma. Their team has developed a novel preparation process for SFNs, based on controlled precipitation. The reported process addresses the production-related challenges of SFNs, offering a method that is faster, has high reproducibility, and has improved scalability.

From the contents of this Special Issue, it is evident that the multidimensional challenges to bioavailability posed by poor drug solubility continue to drive innovation across the field of pharmaceutics. From advanced screening technologies and novel material applications to the strategic use of nanotechnology and amorphous and lipid-based formulations, each featured article provides a glimpse into the potential for significant improvements in dosage form design, manufacture, testing, and performance. These contributions not only reflect the current progress toward overcoming the issues stemming from poor drug solubility, but also highlight the multi-disciplinary efforts being made by pharmaceutical scientists to advance pharmaceutical product development. As we progress on these endeavors, the scientific community will continue to explore and further develop these innovative strategies. Such efforts are bound to streamline drug product development, with the ultimate aim of improving patient outcomes.

